# Chinese liquor extract attenuates oxidative damage in HepG2 cells and extends lifespan of *Caenorhabditis elegans*

**DOI:** 10.1002/fsn3.1564

**Published:** 2020-05-23

**Authors:** Jie Liu, Huailing Wang, Xiaoyu Liu, Guohao Zhang, Zhigang Liu

**Affiliations:** ^1^ Department of Respirology & Allergy Third Affiliated Hospital of Shenzhen University Shenzhen China; ^2^ State Key Laboratory of Respiratory Disease for Allergy at Shenzhen University Shenzhen Key Laboratory of Allergy & Immunology Shenzhen University School of Medicine Shenzhen China

**Keywords:** *Canenohabditis elegans*, chinese liquor, HepG2 cells, homofuraneol, stress resistance

## Abstract

Chinese liquor is obtained from various grains by fermentation and complex processes. Chinese liquor contains complex ingredients. However, the low contents and presence of ethanol restricted the flavor substances function study. In current study, a flavor substance, homofuraneol (HOMO) was isolated from the Chinese liquor and the potency against H_2_O_2_‐induced oxidative damage in HepG2 cells and lifespan‐extending ability in *Caenorhabditis elegans* were explored. Results indicated that HOMO increased the HepG2 cells cytoactive by eliminating excessive intracellular free radicals, upregulating antioxidant enzyme activity and inhibiting the phosphorylation of mitogen‐activated protein kinases (MAPKs) pathway. Further study revealed that HOMO extended the lifespan of N2 nematodes under normal and oxidative stress conditions. Moreover, RT‐PCR results showed that paraquat activated the expression of *PMK‐1* and *SKN‐1* was significantly regulated by HOMO. Of note, our results indicated that HOMO recovered the redox states of HepG2 cells by targeting MAPKs and upregulating the stress resistance of nematodes by modulating the expression of stress‐responsive genes, such as DAF‐16.

## Graphical abstract

We isolated a special flavor substance, homofuraneol (HOMO) and studied its effect against H_2_O_2_‐induced oxidative damage in HepG2 cells and its ability to extend the lifespan of *Caenorhabditis elegans*. The results indicated that HOMO recovered the redox status of HepG2 cells by targeting MAPKs and increased the lifespan and stress resistance of nematodes by modulating the expression of stress‐responsive genes, such as that encoding DAF‐16.

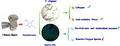

## 1 INTRODUCTION

A variety of damaging processes and diseases, such as carcinogenesis, inflammation, aging, and atherosclerosis are related to overproduction of reactive oxygen species (ROS). Further, excessive ROS would cause oxidative stress, leading to serious damage and/or apoptosis. Therefore, it is of great significance to attenuate oxidative stress. Antioxidant defenses include antioxidant enzymes, such as superoxide dismutase (SOD), catalase (CAT), and glutathione peroxidase (GSH‐Px) (Ighodaro & Akinloye, 2017). At the nonenzymatic level, phytochemicals provide significant protection against oxidative damages (Cheng, Lu, & Yen, 2016). Phytochemicals may exert antioxidant effects against oxidative stress through activating endogenous antioxidant enzymes (Khammanit et al., 2017). As reported, increased oxidative stress may damage macromolecules by triggering mitogen‐activated protein kinase (MAPK) signaling pathways (Xiong, Dong, Wang, Tong, & Li, 2015). MAPKs such as extracellular signal‐regulated kinase (ERK), p38, and c‐Jun N‐terminal kinase (JNK) play significant roles in cell proliferation signaling pathways (Kim et al., 2017). MAPK signaling pathways can also be modulated by ROS, by the expression levels of genes encoding antioxidant enzymes, and by antioxidant enzyme activity (Jiang et al., 2017).

The characteristics of the genome and some important signal pathways are conserved between nematodes and humans, and the human aging process is similar to that of nematodes. Thus, the *Caenorhabditis elegans* is a famous model for studying aging with short lifespan, rapid generation, and experimental flexibility (David et al., 2010; Oh & Kim, 2013). Further, it is also widely used in biomedical and toxicological researches (Jia et al., 2017; Wei, Zeng, Ke, & Wang, 2016).

Studies reported that the correlation between extended lifespan and resistance to oxidative stress is strong. Nematodes with daf‐16 mutation were found to be hypersensitive to oxidative stress and exhibited accelerated aging (Yan et al., 2016). They had mitochondrial ultra‐structural abnormalities resulting in a loss of mitochondrial membrane potential and showed increased apoptosis rates during aging. Similarly, the Y102 nematode with deleted pmk‐1 was found to be more susceptible to oxidative stress (Bolz, Tenor, & Aballay, 2010).

As reported, long‐term alcohol abuse may result in alcoholic liver diseases (Warren & Murray, 2013). The volume and duration of drinking are closely related to alcoholic liver diseases, and the main factor in alcoholic hepatic injury is the acetaldehyde and hydroxyl free radicals oxidized from alcohol, and these hydroxyl free radicals may injure the hepatocytes and cause a lipid peroxidation (Niemelä et al., 1998; Yang, Yang, Wu, Lv, & Li, 2016). The chemicals in Chinese liquor may play protective potency to the liver that are partly related to their ability of alleviating oxidative damage (Markiewiczgórka, Zawadzki, Januszewska, Hombekurban, & Pawlas, 2011). Nevertheless, little is known about the protective potency and mechanisms of the chemicals at the molecular level. In current study, we explore the protect potency of the flavor compounds in Chinese liquor against oxidative stress in HepG2 cells and the lifespan‐extending functions in *C. elegans*.

## 2 MATERIALS AND METHODS

### 2.1 Reagents and nematode strain preparation

Chinese liquor (Moutai liquor, 53% v/v) was acquired from the KweiChow Moutai Distillery. The brand name is mentioned only for accuracy and does not imply any research contact with the manufacturer, nor is an advertisement for the product. The sample was prepared as follows: 50 ml Moutai liquor, 20 ml H_2_O, and 90 ml CHCl_3_ were added to a 250‐ml separating funnel. After extraction, the CHCl_3_ layer was evaporated at 0°C with Termovap Sample Concentrator. Approximately 0.33 mg residue was left and marked as the Chinese liquor extract (CLE). This was stored at −20°C in the dark until analysis. The CLE was analyzed on a HPLC (Agilent 1200 HPLC) equipped with a Zorbax, SB C‐18 column (4.6 × 250 mm, 5 µm). The elution solvent system was composed of water‐trifluoroacetic acid (solvent A; 100:0.1, v/v) and acetonitrile‐trifluoroacetic acid (solvent B; 100:0.1, v/v). The CLE was separated using a gradient elution from 25% to 70% solvent B for 40 min at a flow rate of 1.0 ml/min. The detection wavelength was set at 230 nm, and the column temperature was 20°C. The flavor compound homofuraneol (HOMO) was also isolated by this method.

The transgenic nematode strains CF1038 [*daf‐16* (mu86) I] and *AY102 [pmk‐1 (km25) IV]* were purchased from the *Caenorhabditis Genetics Center*. All nematodes were maintained at 25°C on nematode growth medium (NGM) seeded with *Escherichia coli* OP50 as the food source.

Antibodies against CAT, phosphorylated ERK, phosphorylated JNK, and phosphorylated p38 were purchased from Cell Signaling Technology Inc. Antibodies against SOD, GSH‐Px, and β‐actin were purchased from Santa Cruz Biotechnology, Inc. All the other reagents were of the highest quality available.

### 2.2 HepG2 cell culture and viability assays

HepG2 cells were cultured in DMEM supplemented with 10% fetal bovine serum, 100 IU/ml penicillin, and 100 μg/ml streptomycin at 37°C in a 5% CO_2_ atmosphere.

The antioxidant damage potency of the chemicals isolated from the Chinese liquor was designed as follows: the HepG2 cells were cocultured with the chemicals at 0.020 mg/ml for 24 hr, after that treated with H_2_O_2_ (100 μM) for 12 hr. Then, the cell viability was detected with a CCK‐8 kit.

For the 2,5‐diphenyl‐tetrazolium bromide (MTT) assay, HepG2 cells were seeded in a 96‐well plate and treated with different concentrations of the HOMO, and the MTT was added to each well after the treatments. After 4 hr of incubation at 37°C, the formazan precipitate was dissolved in 150 μl DMSO and the absorbance was measured at 570 nm with a spectrophotometer.

As for the experimental design, the HepG2 cells (3.0 × 10^5^ cells/ml) were treated as follows: H_2_O_2_ [HOMO (0.0 mg/ml)], HOMO‐1 [HOMO (0.007 mg/ml)], HOMO‐2 (0.036 mg/ml)], HOMO‐3 (0.18 mg/ml)] for 24 hr, and then treated with H_2_O_2_ (100 μM) for 12 hr. Cells without H_2_O_2_ treatment were served as the control.

### 2.3 ROS detection in HepG2 cells

HepG2 cells were treated with HOMO as experimental design. The media were removed and replaced with the serum‐free media loaded with dichlorofluorescein diacetate (DCFH‐DA) (Molecular Probes). Images were captured under a fluorescence microscope at identical exposure times. Densitometry analysis was performed using Image‐Pro Plus 6.0 software.

### 2.4 Western blotting

HepG2 cells were plated in 6‐well culture dishes, grown to confluence, and treated with HOMO for 24 hr. After incubation, cells were washed with ice‐cold PBS, scraped, pelleted, and lysed in radioimmunoprecipitation assay (RIPA) buffer (protease inhibitor cocktail and phosphatase inhibitor). After incubation for 1 hr on ice, cell lysates were centrifuged (3,000 *g* × 30 min). Lysate protein concentrations were detected by a BCA protein assay kit (Thermo Scientific), and the lysates were adjusted with lysis buffer. Proteins were resolved by SDS‐PAGE on a 10%‐15% acrylamide gel and transferred onto immobilon polyvinyl difluoride (PVDF) membranes. The blots were blocked with blocking buffer for 10 min at room temperature and then probed with antibodies for 1 hr at room temperature. Then, the blots were incubated with a peroxidase‐conjugated secondary antibody (1:3,000 dilutions) for 1 hr at room temperature. Densitometry analysis was performed using Image‐Pro Plus 6.0 software.

### 2.5 Preparation of *Caenorhabditis elegans* and safety assays

Age‐synchronized populations of L4‐larvae nematodes were obtained as reported (Qiao et al., 2014). HOMO was added to the NGM plates in a final concentration of 0.20 mg/ml before plating. Gentamicin (30 mg/ml) was added to the NGM plates to inhibit the microbial contamination. The N2 nematodes were maintained at 25°C on NGM seeded with *E. coli* OP50.

For the safety assessment, lethality, growth, brood size, locomotion behavior, and intestinal reactive oxygen species (ROS) production were tested as reported (Zhuang et al., 2014). The HOMO treatments were performed for 4 days from the L4‐larval stage.

### 2.6 Nematode stress resistance assays

The oxidative stress assay was designed as follows: the synchronized young adult nematodes were grown on NGM seeded with *E. coli* OP50 and treated with HOMO‐1 (HOMO, 0.025 mg/ml), HOMO‐2 (HOMO, 0.10 mg/ml), HOMO‐3 (0.40 mg/ml), respectively, for 4 days. After that, the nematodes were transferred to the NGM plates containing 1 mM paraquat for 12 hr. Then, the nematodes were transferred to the NGM plates (day 0), and the survival was monitored daily (≥80 nematodes per group). Nematodes that did not move when gently touched with a soft wire were considered as dead (Lithgow & Walker, 2002). All the trials were repeated at least three times.

### 2.7 ROS detection in nematodes treated with HOMO

Synchronized nematodes were placed on NGM plates, subjected to HOMO‐1 (HOMO, 0.025 mg/ml), HOMO‐2 (HOMO, 0.10 mg/ml), and HOMO‐3 (0.40 mg/ml) for 4 days, respectively, and then, treated with paraquat (1 mM) for additional 8 hr. After that, the nematodes were collected and washed with M9 buffer, lysed with double distilled water in ice bath. Nematodes were prepared for measurement of intracellular ROS levels by the ROS‐specific fluorescent probe DCFH‐DA as described (Wang et al., 2017).

### 2.8 Quantitative real‐time PCR

The nematodes were treated as *Nematode Stress Resistance Assays* described. Then, the total RNA was isolated from about 1,500 nematodes by Trizol reagent (Morimoto et al., 2003). The primer sequences for RT‐PCR are listed in Table 1, and the results are presented as mean ± *SEM* (*n* = 3).

**TABLE 1:** The primer sequences for RT‐PCR (*Caenorhabditis elegans*)

Gene	Direction	Primer sequences (5′−3′)
*sir‐2.1*	Forward	ACTGAGATGCTCCATGACAATAAG
	Reverse	GCAAGACGAACCACACGAAC
*age‐1*	Forward	CCTGAACCGACTGCCAATC
	Reverse	GTGCTTGACGAGATATGTGTATTG
*akt‐1*	Forward	AAAGCCTAAGGAAGGACAACC
	Reverse	ACATGACCACTCCGACTCCC
*daf‐16*	Forward	TCAAGCCAATGCCACTACC
	Reverse	TGGAAGAGCCGATGAAGAAG
*skn‐1*	Forward	CACGCCGTCAGCGAAGTA
	Reverse	GAATCCAACGCTGACGAAC
*pmk‐1*	Forward	TGTATGGTCAGTTGGGTGTATTC
	Reverse	CCTCATCTTCCCTCTTCGTCAG

## 3 RESULTS AND DISCUSSION

### 3.1 Protective effects of HOMO on HepG2 Cells

After we got the CLE, further experiments were performed with the high‐performance liquid chromatography and gas chromatography–mass spectrometry. Finally, twenty‐six chemicals were identified and the results of antioxidant activity showed that 2‐ethyl‐4‐hydroxyl‐5‐methyl‐3(2H)‐furanone (Homofuraneol) performed the strongest protective effect. The MS and NMR data were listed as follows: the molecular mass of the compound was determined to be 142.06 Da. The NMR data were as follows: ^1^H NMR (CDCl_3_, 400 MHz) *δ*: 10.5 (1H, s, OH), 4.21 (1H, t, *J* = 1.42 Hz, H‐1), 2.23 (3H, s, ‐CH_3_), 1.72 (2H, td, *J* = 1.42 and 0.30 Hz, ‐CH_2_), 0.89 (3H, t, *J* = 0.30 Hz, ‐CH_3_); ^13^C NMR (CDCl_3_, 100 MHz) *δ*: 203.1 (C‐2), 142.3 (C‐3), 130.1 (C‐4), 82.6 (C‐1), 21.6 (C‐1′), 17.8 (C‐1″), 9.3 (C‐2′).

The cytotoxicity of HOMO to HepG2 cells was detected by MTT assay, and the results showed that the HOMO performed no cytotoxicity to the HepG2 cells upto 0.180 mg/ml. As reported, the Chinese liquor is an extremely mixture, and it's rich in unsaturated alcohols, esters, and amino acids (Yao et al., 2015). Further, the flavor substances in Chinese liquor may perform excellent biological activities, such as antithrombosis potency (tetramethylpyrazine) (Smith et al., 2005).

### 3.2 HOMO reduced ROS and activated antioxidant enzymes in HepG2 cells

Excessive ROS accumulation would harm the cellular function and cause diseases (Pashkovskaia, Gey, & Rödel, 2018). H_2_O_2_ is a major component of ROS, and it may cause oxidative damage to the physiological processes and result in a range of pathologies (Prasad, Gupta, & Tyagi, 2016). Hence, the H_2_O_2_ is commonly used as an experimental agent to investigate the mechanism of cell injury induced by the oxidative stress (Ma et al., 2016).

**FIGURE 1:**
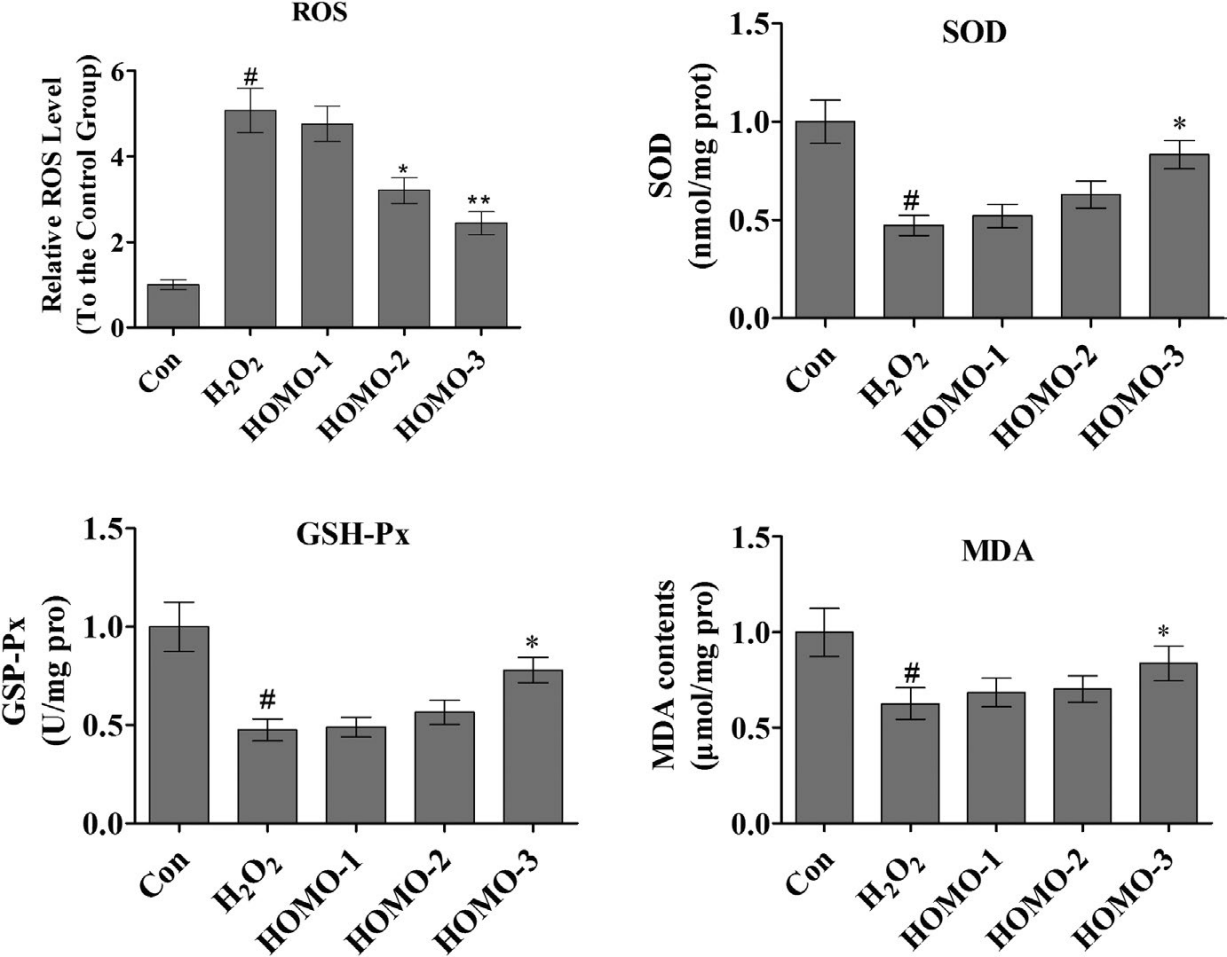
The levels of ROS, SOD, CAT, and GSH‐Px with HOMO treatments. HOMO represses intracellular ROS production under H_2_O_2_ stimulation in HepG2 cells. HOMO affects the SOD, CAT, and GSH‐Px levels in H_2_O_2_‐induced HepG2 cells. HepG2 cells were pretreated with HOMO for 24 hr and then stimulated with H_2_O_2_ for 12 hr. ROS, SOD, CAT, and GSH‐Px productions were measured as described. H_2_O_2_ [HOMO (0.0 mg/ml)], HOMO‐1 [HOMO (0.007 mg/ml)], HOMO‐2 (0.036 mg/ml)], HOMO‐3 (0.18 mg/ml)] for 24 hr, and then H_2_O_2_ (100 μM) for 12 hr. Cells without H_2_O_2_ treatment were used as the control group. Data are represented as the mean ± *SEM* (*n* = 3). ^*^ *p* < .05, ^**^ *p* < .01, versus H_2_O_2_, ^#^ *p* < .05, ^##^ *p* < .01, versus control, indicate statistically significant difference

To investigate the protective effect of HOMO, we measured the ROS level and antioxidant enzyme activities in HepG2 cells under H_2_O_2_ stimuli (Figure 1). The ROS level in the control group was set as 1. The relative ROS level in the H_2_O_2_ group was increased by 5.01 folds. Interestingly, HOMO markedly inhibited the ROS production. After the HOMO treatments, the relative ROS levels were 4.76 ± 0.41 (HOMO‐1), 3.21 ± 0.30 (HOMO‐2), and 2.44 ± 0.27 (HOMO‐3), respectively. Study revealed that the phytochemicals can strongly inhibit the oxidation activities of scavenging peroxyl radicals (Morita, Naito, Niki, & Yoshikawa, 2017). In current study, HOMO decreased the accumulation of ROS in HepG2 cells in a dose‐dependent manner. These results confirmed that the HOMO could reduce intracellular oxidative stress induced by H_2_O_2_ and protect HepG2 cells from oxidative stress damage.

Intracellular antioxidant enzymes play a significant role of protecting cells against oxidative stress. The activity changes of these enzymes can be considered as a bio‐marker of antioxidant response to oxidative stress. We explored the activity of these enzymes after HOMO treatments to determine whether the HOMO performs antioxidant function by stimulating the activity of antioxidant enzymes, as well as directly scavenging ROS/free radicals. When cells were treated with H_2_O_2_ (100 μM) alone, the activities of CAT, GSH‐Px, and SOD were sharply decreased (Figure 1). However, the H_2_O_2_‐treated cells treated with 0.007 mg/ml HOMO showed 15.3%, 45.2%, and 40.9% increases in CAT, SOD, and GSH‐Px activity, respectively, those treated with 0.18 mg/ml HOMO showed 33.7%, 75.9%, and 64.0% increases in CAT, SOD, and GSH‐Px activity, respectively.

### 3.3 Effects of HOMO on expression of antioxidant enzymes and phosphorylation of MAPK

The antioxidants can regulate both the content and the expression levels of antioxidant enzyme encoding genes (Mueller, Blum, Kluge, & Mueller, 2012). Therefore, we further explored whether HOMO can stimulate the expressions of antioxidant protein.

**FIGURE 2:**
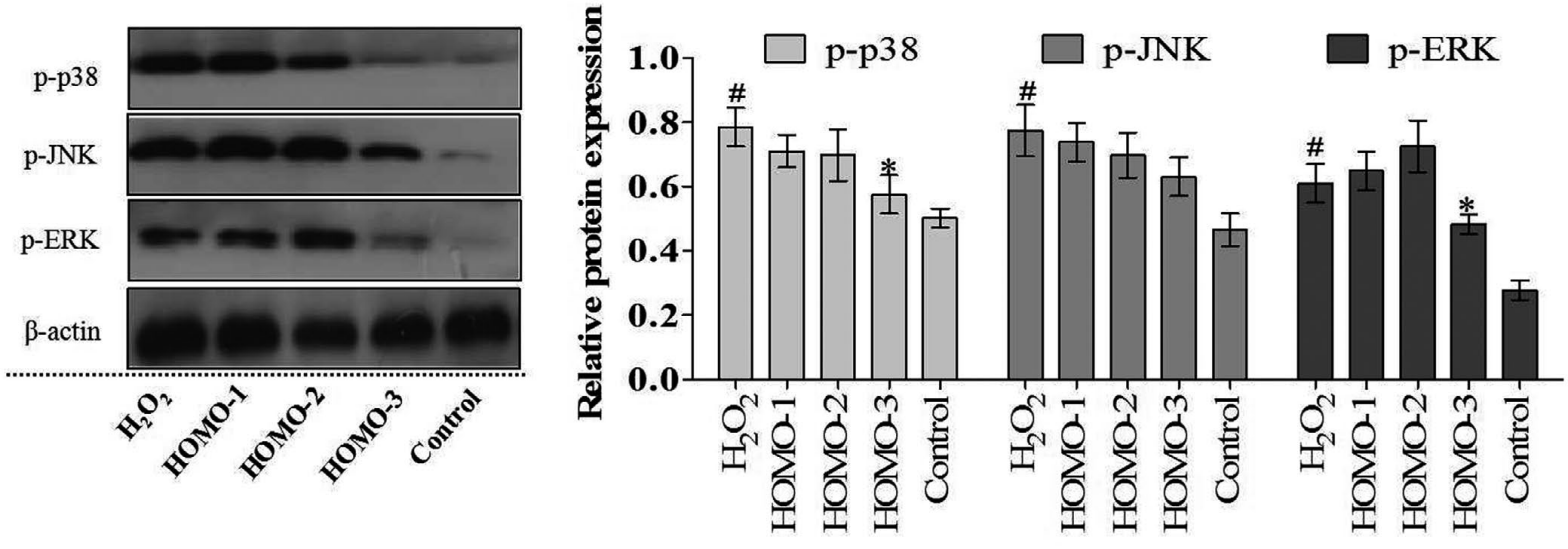
Effect of HOMO on proteins of phosphorylation of MAPK. H_2_O_2_ [HOMO (0.0 mg/ml)], HOMO‐1 [HOMO (0.007 mg/ml)], HOMO‐2 (0.036 mg/ml)], HOMO‐3 (0.18 mg/ml)] for 24 hr, and then H_2_O_2_ (100 μM) for 12 hr. Cells without H_2_O_2_ treatment were used as the control group. The protein expression levels were measured using western blot. The density of each lane was presented as mean ± *SEM*. (*n* = 3). Blots were quantified using ImageJ software. (*n* = 3). ^*^ *p* < .05, ^**^ *p* < .01, versus H_2_O_2_, ^#^ *p* < .05, ^##^ *p* < .01, versus control, indicate statistically significant difference

The expression of protective proteins may be induced by excessive ROS through cellular signal transduction pathways. Studies reported that ROS can activate the Nrf2 through the mitogen‐activated protein kinase (MAPK) pathway to protect against oxidative stress (Jang & Cho, 2016). To determine whether antioxidant enzyme expression induced by HOMO was associated with the MAPK pathway, we examined the phosphorylation levels of three MAPKs; ERK, JNK, and p38. As shown in Figure 2, the phosphorylation of MAPKs was markedly upregulated with H_2_O_2_ stimuli. Interestingly, HOMO treatment decreased the H_2_O_2_‐stimulated phosphorylation of MAPKs. With 0.18 mg/ml HOMO treatment, the p‐ERK, p‐JNK, and p‐p38 levels were decreased by 20.8%, 18.6%, and 26.7%, respectively. Here, the results also indicated that the HOMO performed protective effects against the oxidative damage. The results indicated that HOMO significantly attenuated H_2_O_2_‐induced damage and activated the MAPKs.

The activity and expressions of antioxidant enzyme may modulated by the upstream MAPKs such as JNK, ERK and p38. The phosphorylation of the MAPKs has been shown to be mediated through H_2_O_2_‐induced oxidative stress (Kang et al., 2010). Our results showed that HOMO decreased the phosphorylation of MAPKs in a dose‐dependent manner. To the best of our knowledge, this is the first report that HOMO can modulate MAPKs. Our data demonstrated that HOMO protected cells against the oxidative damage by scavenging ROS and increasing the activity of cellular antioxidant enzymes via the inhibition of MAPKs.

Both the antioxidant enzymes and antioxidants play significant roles in reducing oxidative stress damages (Kong et al., 2016). The phytochemicals have been reported to have excellent antioxidant and ROS‐scavenging abilities (Peng, Lee, & Lim, 2017). Chinese liquor, one of the most famous wines, is a staple beverage worldwide with a special flavor and centuries of history (Dahe, Tiandao, & Guohong, 2011). Oxidative stress is one of the main factors in diabetic complications, and results from ROS generation in the liver, which in turn causes hepatic insulin resistance (Wang et al., 2014). As well as having direct antioxidant properties, some compounds may also activate intracellular signaling pathways like the MAPK pathway to prolong the cellular defense response. Antioxidant enzymes such as SOD, CAT, and GSH‐Px are regarded as the first line of the antioxidant defense system against ROS. Our results showed that HOMO depressed the ROS levels and increased the activity of these antioxidant enzymes.

### 3.4 *Caenorhabditis elegans* safety assays

We tested the safety of HOMO by exposing L4‐larvae nematodes to HOMO for 24 hr. Treatment with 0.80 mg/ml HOMO did not induce lethality, influence development, or affect the brood size or locomotion behavior. These results suggested that the nematodes were not harmed by HOMO at a concentration of 0.80 mg/ml. Previous studies have shown that proliferating bacteria can harm nematodes by producing deleterious metabolites (Garigan et al., 2002). Considering the antimicrobial properties of the HOMO, it was possible that HOMO inhibited the growth of *E. coli* OP50 under the experimental conditions and extended the lifespan of nematodes. To avoid this, we cultured *E. coli* OP50 in LB medium with HOMO in different concentrations. The growth of *E. coli* OP50 was not inhibited up to 0.40 mg/ml. Therefore, it was unlikely that HOMO prolonged the lifespan through its antimicrobial effect.

### 3.5 HOMO extended the lifespan of N2 nematodes

**FIGURE 3:**
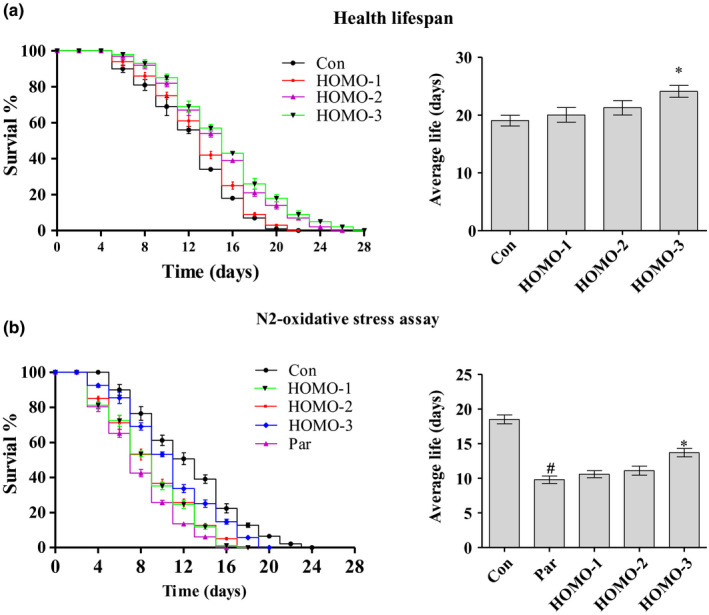
Effects of HOMO on N2 nematodes lifespan under normal or oxidative stress conditions. (a) The young adult nematodes were cultured at 25°C with HOMO for 4 days and then transferred to the NGM plates, and the survivals was monitored every day (*n* ≥ 80 animals). The experiment was repeated multiple times until a representative trial is shown. (b) Synchronized young adult nematodes were grown in NGM with HOMO: Par (HOMO, 0.0 mg/ml), HOMO‐1 (HOMO, 0.025 mg/ml), HOMO‐2 (HOMO, 0.10 mg/ml), HOMO‐3 (0.40 mg/ml), FUDR (50 μg/ml), and *Escherichia coli OP50* as the food source. Nematodes pretreated as described above for 4 days, and then the nematodes were transferred onto NGM plates containing 1 mM paraquat for 12 hr, after that, the nematodes were transferred to the NGM plates and take the day as day 0. All trials were repeated at least 3 times, and the survival nematodes were counted every day, *n* ≥ 80 nematodes each group. Nematodes that did not move when gently touched with a soft wire were considered dead. Data are presented as the mean ± *SEM*. ^*^ *p* < .05, ^**^ *p* < .01, versus H_2_O_2_, ^#^ *p* < .05, ^##^ *p* < .01, versus control, indicate statistically significant difference

Studies have revealed the close relationship between oxidative damage and aging (Panich, Sittithumcharee, Rathviboon, & Jirawatnotai, 2016; Ruiz, Cabrera, López‐Jiménez, Zamora, & Pérez‐Llamas, 2017). Phytochemicals with antioxidant properties, such as flavonoid, perform excellent antiaging activities (Liu & Xing, 1990). In current study, the HOMO reduced oxidative damage and improved the survival rate of HepG2 cells. To investigate whether HOMO also has a protective effect in vivo, we detected its antiaging effects on nematodes. Treatments with HOMO extended the average lifespan of the N2 nematodes by 5.19% (HOMO‐1), 11.7% (HOMO‐2), and 26.6% (HOMO‐3) (*p* < .05), compared with the control group (Figure 3a). These data indicated that HOMO has strong antiaging properties in the animal model.

### 3.6 Oxidative stress resistance effects of HOMO on nematodes

Mutations in the insulin/IGF‐I‐like signaling pathway were shown to lead to resistance to a variety of stresses, including oxidative stress induced by paraquat (Sampayo, Olsen, & Lithgow, 2003). Under oxidative stress conditions, HOMO functioned as a powerful antioxidant with the capacity to alleviate cellular damage. This explained why HOMO extended the lifespan of nematodes.

To investigate whether HOMO can induce stress resistance in nematodes, N2 nematodes were pretreated with the HOMO for 4 days before exposure to the oxidative stress. As is shown in Figure 3b, the HOMO performed a significant protective effect in N2 nematodes. The HOMO treatments extended the lifespan from 9.80 ± 0.53 days (Paraquat group) to 10.6 ± 0.51 days (HOMO‐1), 11.1 ± 0.65 days (HOMO‐2), and 13.7 ± 1.62 days (HOMO‐3), corresponding to an extension of the mean lifespan by 8.20%, 13.3%, and 41.6%, respectively.

The aging is a complex process, which is influenced by both environmental and genetic factors (Bloss, Pawlikowska, & Schork, 2011). Nematodes are a popular model for studying aging and longevity with the properties of short lifespan, fully sequenced genome, and experimental flexibility. In addition, the nematodes share high conservation of biochemical pathways and similar aspects of aging with the human (Jenkins & Allen, 2014). As the results list in Figure 3, HOMO showed a powerful antioxidant capacity and extended the lifespan of nematodes both in normal and oxidative stress conditions.

### 3.7 HOMO reduced relative ROS levels in nematodes under oxidative stress

**FIGURE 4:**
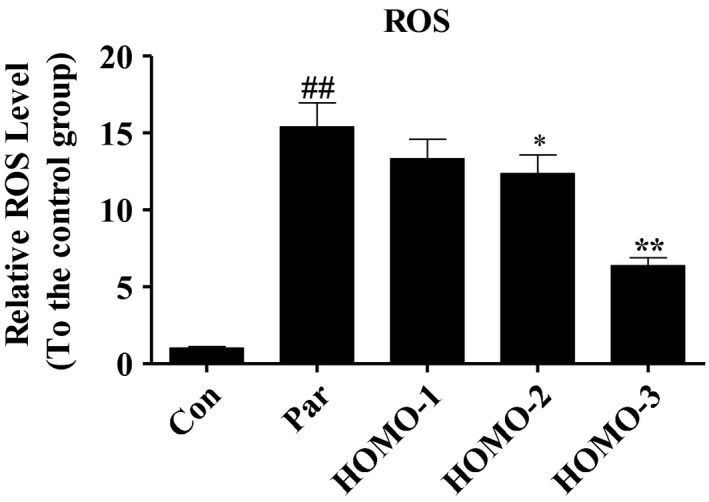
The relative level of ROS in N2 nematodes with paraquat stimuli. Synchronization nematodes were plated at NGM plates which contained HOMO as described above. After the treatment for 4 days and paraquat (1 mM) for 8 hr, all nematodes were collected and lysed with double distilled water in ice bath after washed with M9 buffer. The intracellular ROS was detected with the ROS‐specific fluorescent probe DCFH‐DA. Data are presented as the mean ± *SEM*. ^*^ *p* < .05, ^**^ *p* < .01, versus H_2_O_2_, ^#^ *p* < .05, ^##^ *p* < .01, versus control, indicate statistically significant difference

The ROS are generated as by‐products in normal or irregular cells and systems and play important roles in many processes. Excessive ROS may cause a serious fatal damages to the cells, while antioxidants can detoxify ROS and ameliorate the damage (Kim & Wong, 2013). Here, we measured the ROS level in nematodes using the DCFH‐DA staining method (Zhou, Tan, Zhang, & Wu, 2017). As shown in Figure 4, the paraquat stimuli resulted in a 15.4‐fold upregulation in ROS level. However, the HOMO decreased the ROS levels by 13.6% (HOMO‐1), 19.5% (HOMO‐2), and 58.6% (HOMO‐3), respectively.

### 3.8 Genetic requirements for increased stress resistance in HOMO‐treated nematodes

**FIGURE 5:**
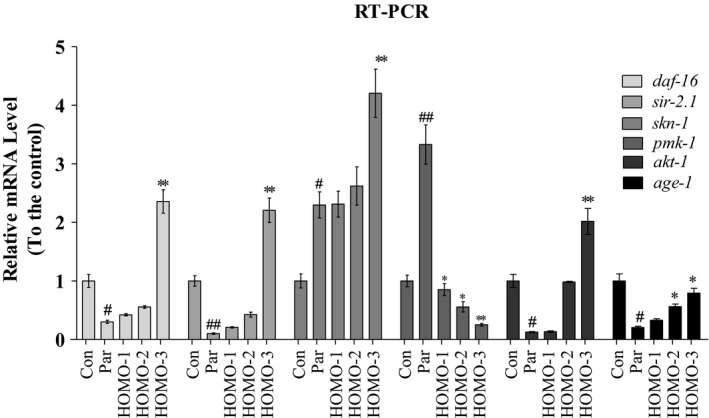
The relative expression of mRNA in N2 nematodes with paraquat stimuli. Synchronization nematodes were plated at NGM plates which contained HOMO as described. After the treatment for 4 days and paraquat (1 mM) for 8 hr, all nematodes were collected and lysed with double distilled water in ice bath after washed with M9 buffer. The relative expression level of mRNA including: *daf‐16*, *sir‐2.1*, *skn‐1, pmk‐1*, *akt‐1,* and *age‐1* in *Caenorhabditis elegans*. Assay results were confirmed by RT‐PCR, and the relative expression levels of the mRNA were determined. ^*^ *p* < .05, ^**^ *p* < .01, versus H_2_O_2_, ^#^ *p* < .05, ^##^ *p* < .01, versus control, indicate statistically significant difference

Studies demonstrated that the AKT‐1 and DAF‐16/FOXO are significant factors in the insulin/IGF‐1 pathway, which is associated with longevity in nematodes (Warnhoff et al., 2014). In this study, HOMO failed to extend the lifespan of the *daf‐16* mutant, suggesting that DAF‐16 may be one of its targets. PMK‐1/p38 phosphorylates SKN‐1 directly to activate its activity, and the p38 MAPK pathway is also strongly related to the oxidative stress response (Lim et al., 2012). In this study, AY102 nematodes (a deleted *pmk‐1* strain) were more susceptible to oxidative stress than were wild‐type nematodes. The AY102 nematodes treated with HOMO could not recover from oxidative damage (results not shown), indicating that HOMO may modulate PMK‐1 activity. However, further research on the exact mechanism is necessary. As shown in Figure 5, the expression levels of the genes *sir‐2.1*, *daf‐16*, *akt‐1*, and *age‐1*, which are closely related to aging or oxidative resistance, were all recovered by HOMO treatments. These findings indicated that HOMO exerted the protective effects may by modulating the expression of stress‐responsive genes including *daf‐16* and *pmk‐1*.

## 4 CONCLUSION

A flavor compound from Moutai liquor, HOMO, significantly attenuated damage induced by H_2_O_2_ and activated MAPKs in vitro. Further, HOMO extended the lifespan of nematodes by activating DAF‐16/FOXO and PMK‐1/p38, which are related to longevity and stress resistance. These results provided evidence for the mechanisms of HOMO in cytoprotection and antiaging, and imply that antioxidants in Chinese liquor may be beneficial for human health. However, an enormous amount of researches showed the Chinese liquor was harmful to the animal models. Here, we also suggest that keep away from the alcoholic drink. Further, our research showed the value of using simple biological models to screen compounds that have potential applications in food, nutraceutical, and pharmacological industries.

## CONFLICT OF INTEREST

The authors declare no conflicts of interest.
